# Exploring life with autism: Quality of Life, daily functioning and compensatory strategies from childhood to emerging adulthood: A qualitative study protocol

**DOI:** 10.3389/fpsyt.2022.1058601

**Published:** 2022-11-25

**Authors:** Elisabeth Øverland, Åshild Lappegard Hauge, Stian Orm, Elizabeth Pellicano, Merete Glenne Øie, Erik Winther Skogli, Per Normann Andersen

**Affiliations:** ^1^Division of Mental Health Care, Innlandet Hospital Trust, Brumunddal, Norway; ^2^Department of Psychology, Inland Norway University of Applied Sciences, Lillehammer, Norway; ^3^Department of Psychology, University of Oslo, Oslo, Norway; ^4^Department of Clinical, Educational and Health Psychology, University College London, London, United Kingdom; ^5^Macquarie School of Education, Macquarie University, Sydney, NSW, Australia; ^6^Research Department, Innlandet Hospital Trust, Brumunddal, Norway

**Keywords:** autism, Quality of Life, emerging adulthood, compensatory strategies, autistic burnout

## Abstract

**Introduction:**

This study aims to investigate self-perceived quality of life, daily functioning, and the use of compensatory strategies in emerging adults with autism^1^.

**Methods and analysis:**

Participants will be recruited from the Lillehammer Neurodevelopmental 10-year follow-up study (LINEUP), with the aim of 15 individual in-depth interviews. Subsequently, two focus groups with clinicians will be invited to reflect on the themes found in the individual interviews. All interviews will be recorded and analyzed using reflexive thematic analysis.

**Ethics and dissemination:**

The study is approved by the Regional Committee for Medical Research Ethics in South-East Norway. The findings will be disseminated to academic and clinical audiences through journal articles and conference presentations. To reach the broader autistic and autism communities, the findings will be shared with the Autism Society at national and local meetings, in their membership magazine, and on their social media channel.

## Introduction

In this study, we seek to explore how emergent adults with autism experience, describe and reflect on what constitutes a good Quality of Life (QoL) and how they set out to achieve it. Emerging adulthood is described as the period of life between adolescence and young adulthood (i.e., 18–25 years) (1). This period is characterized by identity exploration, instability, self-focus, and a feeling of being in-between adolescence and an adult. It is also associated with greater levels of depression and suicidality (2). A handful of studies have looked at the transition process from adolescence to emerging adulthood in those with autism (3–6). Some of these studies, particularly those taking a qualitative approach, have highlighted the disconnect between the traditionally defined successful outcomes and the actual perceived quality of life for people with autism (5, 6) and have questioned the use of normative measures to describe quality of life or wellbeing. There is therefore an imperative to understand how people with autism perceive a good life themselves, rather than one constructed by others (7, 8).

We know that young people with autism probably are likely to encounter often substantial obstacles in their transition to adult life, compared to their neurotypical peers. In a review of qualitative studies, Anderson et al. (3) found that poor transition outcomes for young adults with autism seemed connected to several intersecting factors, including poor person-environment fit, uncertainty about the role of parents during transition, and a lack of comprehensive or poorly administered services. Matthews et al. (9) found that the issue of living interdependently was influenced by the challenge of combining the ability to manage daily life obstacles on one's own with support from formal services, and parents' involvement. Their study also revealed large intra-individual variability regarding self-reported strengths and difficulties in everyday function. Cribb et al. (5) emphasized the importance of letting young people with autism be in control of their own lives, with identity building and personal autonomy as crucial elements for well-being.

Building upon earlier studies, in this study, we seek to gather in-depth experiences, perceptions, and descriptions concerning QoL, and how different aspects of autistic features influence the perceived QoL for emerging adults with autism. Here, we share our protocol, to inform others about the study, share its tools and, at the same time, highlight key conceptual issues driving this project—including Quality of Life and related factors such as compensatory strategies and autistic burnout. We present a brief overview of each of these issues below.

### Quality of Life (QoL)

Quality of Life refers to an individual's subjective perception of the quality of their health, relationships, school/job satisfaction, and participation in society (10). Adults with autism seem to have a poorer QoL throughout their lifespan when compared to neurotypical adults, and when measured with instruments designed for the general population (11, 12). Intrapersonal factors connected to autism may affect QoL negatively, such as high levels of stress, sleep problems, sensory processing sensitivities, executive function difficulties and vulnerability for developing mental health problems (13–17). Interpersonal factors such as social interaction challenges might lead to social withdrawal and isolation and a reduced QoL (18). Young people with autism are also at much higher risk of experiencing social exclusion and bullying at school (19, 20) and past experiences of being bullied is a predictor of poorer perceived QoL (15). Autism is often understood by others within a deficit narrative (21, 22), which can have a deleterious impact on their opportunities. Environmental factors include the often-severe lack of support they receive within education, health and care; Crane et al. (23) found that young people with autism experience high levels of stigma and often face severe obstacles when they try to access mental health support. Many adults with autism experience lack of understanding of autism by healthcare staff and little coherence during transitions from one life-phase to another (e.g., from student to employee) (24, 25).

Reassuringly, several studies have shown that a good QoL is possible for people with autism, when the right conditions have been met (17, 24). A more positive QoL amongst adults with autism is connected to being employed, receiving support and being in a close relationship (24). Many characteristics of autism can also be regarded as strengths, both in and of themselves, and often depending upon the context. The focus on context underlines the importance of person-environment fit—in which recognition of possibilities, and modifications in society to be more “autism-friendly”, might build a more positive identity and create a more inclusive attitude amongst people with autism (24, 26, 27). How people with autism perceive their QoL could therefore depend on what kind of context in which they find themselves.

### Measurements of Quality of Life

As outlined in section Quality of Life (QoL), several challenges have been identified in the lives of persons with autism and have been found to be important for QoL. However, there is no general agreement on what constitutes a good QoL for people with autism (11). QoL may be perceived as something different for people with autism than for people in the general/neurotypical population, and hence traditional measures of QoL may not sufficiently tap into what QoL consists of for people with autism (26).

During the last two decades, report scales have been designed specifically to measure the QoL for people with disabilities, and these have been used in studies measuring QoL in children, adolescents and adults with autism (28–31). McConachie et al. (26) found that some topics highly relevant for people with autism were not included in the well-used WHO QoL questionnaires developed for the general population. They identified 11 themes relevant for QoL from group interviews with adults with autism in different countries: public knowledge and acceptance of autism, external support and services, financial resources, family support, sensory issues, daily hassles/barriers, autistic identity, self-determination/autonomy, mental health, social engagement, and friendship. This study led to the development of a supplementary QoL item pool, the Autism Spectrum QoL (ASQoL), to be used together with the WHOQOL-BREF and WHO Disability module to capture the specific autism-relevant aspects of QoL (32). The ASQoL form has not yet yielded satisfactory psychometric results (33), however, but further research using adapted forms or scales such as this should reveal more insight into the perceived quality of life for the autistic population.

Nevertheless, all of these quantitative studies on QoL still adopt a normative approach (7). This might not fit the actual lived experiences of people with autism, which cannot be captured through questionnaires that have been developed by and for people without autism. This is unsurprising, give that autism research has historically had little focus on autistic people's individual resilience and positive well-being (8, 22). People with autism have had little involvement in the research itself, including research on the construct of QoL (34–36). The participants in this study will therefore be given the opportunity to bring forth the varied themes and challenges that they experience as central to their lives. We believe that oral, qualitative descriptions can capture more personal experiences than questionnaire-based measures and give us richer insights into the lives of emerging adults with autism.

### Compensatory strategies and QoL

The use of compensatory strategies is common among individuals with autism (37). Common compensatory strategies are masking (or camouflaging) of social cognitive difficulties, learning and following social scripts, and mimicking neurotypical people's behavior in different interpersonal settings (38). The deployment of compensatory strategies is often associated with having a good outcome (i.e., appearing more “neurotypical” for others, being successful, having friends), being diagnosed in adulthood, or being autistic and female. These strategies may appear as both cognitively simple or complex processes and are influenced by the environment in which they appear (39).

Unfortunately, using compensatory strategies can be cognitively taxing and exhausting—and might well lead to a reduced QoL despite otherwise seemingly good functioning (40, 41). Higher executive function skills are associated with greater use of compensatory strategies, but also with less good self-perception, negative emotions and attitudes (38). Mental health problems that arise from increased stress, feeling of inadequacy, shame, anxiety, depression and autistic burnout are also associated with the use of compensatory strategies (24, 37, 38, 40–42). Significantly more women appear to use compensatory strategies than men (38, 43), although the outcome of compensatory strategies is reported to be more positively perceived by men (37). At the same time, camouflaging as a compensatory strategy has been associated with more depressive symptoms in men than in women (38). The negative effects of masking and other compensatory strategies can be understood within a broader sociohistorical context. Pearson and Rose (21) argue that masking is often a response to stigma and marginalization—and that it is of little use to differentiate the impact of masking when it comes to gender, both considering the large number of nonbinary people with autism, and that these differences are probably driven by social context and gendered socialization. Seen in this way, masking could be an unconscious response to society's perception of autism as a disability that should be “fixed”. Indeed, interventions toward autism have focused on minimizing autistic traits, whether harmful or not, and with little focus on how this can influence the autistic identity negatively (44). The prejudice toward autism in society might therefore lead people to hide their autistic traits, to avoid stigma.

### Autistic burnout and QoL

Another aspect severely affecting QoL for many people with autism is “autistic burnout”. Autistic burnout refers to a severe condition of fatigue accompanied by social withdrawal, cognitive dysfunctions and exacerbation of autistic traits. It appears to be distinct from depression and job-related burnout (45). Autistic burnout may seriously impair QoL and seems to be closely preceded by lack of person-environment fit, cognitive stress, and the strain of using compensatory strategies. Again, autistic burnout is linked to the discrimination, lack of facilitation and stigma that people with autism experience while meeting the unaccommodating environment in which they live (41, 45).

School may be one such unaccommodating environment. It is well established that school can be a very challenging time for students with autism. They have a much higher risk of having periods of prolonged absence from school and developing school refusal behavior, in comparison to neurotypical students (46, 47). They often need to deal with significant sensory and social overwhelm, and numerous transitions throughout the day, and can find it challenging to keep up with academic work, especially without the requisite support (48, 49). For some, the challenges will result in a severe condition of fatigue as described in autistic burnout (41, 45). We are interested in if and how our participants describe different ways of experiencing overwhelm and possibly signs of burnout, and if this is connected to autistic traits, compensatory strategies, and/or other social, environmental and personal factors.

### Study aim

In this study, we seek to explore how emerging adults with autism without intellectual disabilities, experience this period of life. How do they describe and conceptualize QoL? How do they describe the influence different features of autism have had on their QoL? Which specific experiences during childhood and adolescence do they think has affected their current QoL? How is their QoL affected by daily activities? If compensatory strategies are used, how do they affect QoL? The study also aims to explore how experienced clinicians reflect upon the stories that are being told. Do the perspectives that derive from the individual interviews challenge or coincide with the view that clinicians already have? Could clinicians gain more knowledge about emerging adults with autism and their experience of QoL based on the themes derived from the individual interviews?

### Objectives

1. To develop knowledge on how emerging adults with autism experience and describe:

a) important elements for Quality of Life in emerging adulthood;
b) different experiences during childhood and adolescence which might have influenced the Quality of Life in emerging adulthood;
c) the challenges and barriers they meet in their everyday life;
d) the ways in which they might consider having autism is an advantage; and
e) the degree to which compensatory strategies are used in social interactions, and the perceived impact of such strategies.

2. To develop knowledge on how people with autism's lived perspective on their own Quality of Life:

a) challenges or coincides with professional health services' view on autism;
b) can inform health, educational and/or occupational services concerning autism.

## Method

### The scientific-philosophical basis for the research project

The analytic approach for this qualitative study will follow reflexive thematic analysis (see section Data analysis). Such studies cannot be conducted in an epistemological and ontological vacuum (50). Instead, as researchers, we recognize—in line with contextualist epistemology—that the knowledge produced cannot be separated from the knower, and the researcher will form part of that knowledge (50). Language is therefore understood as intentional, not as a mirror of an informant's life world, but as an informant's unique perspective on reality.

In our project, we will adopt a critical realism approach, which does not assume that the stories the participants share, are direct reflections of what goes on in the world (51). The data we collect are instead interpreted to determine what underlying structures may lie beneath the shared experiences. This brings in the commitment to ontology, in addition to epistemology (52). Ontology enhances the importance of reflexivity; for us, as researchers, to be aware of our pre-existing assumptions and how these influence the interpretation of our findings (50, 53, 54). Critical realism is not merely data-driven, but uses a more theory- and researcher-driven analytical process (55). Being a qualitative study, the research questions that have been formulated can still be further adapted as the research progresses (56).

### Sample and recruitment

The informants for the individual interviews will be recruited from the Lillehammer Neurodevelopmental Follow-Up Study (LINEUP) in Norway. LINEUP is a 10-year follow-up study on children (mean age at inclusion was 12 years) diagnosed with autism, ADHD, and/or Tourette's Syndrome investigating cognitive and emotional development across three-time points: T1, T2 (two years after intake) and T3 (10 years after intake). Thus far, LINEUP has collected quantitative data at all time points. This study represents the first time that qualitative data will also be collected from these participants.

We will do purposive sampling (57) from the group of emerging adults who were diagnosed within the autism spectrum at T1 (*N* = 38, age range 20–29). Within this group, we aim for the adults that were still displaying sufficient autistic traits to meet diagnostic criteria at T3, 2 years ago. This assessment process, as part of the LINEUP study, is described more thoroughly in the article by Orm et al. (58). There was a minority of women in the inclusion at T1 (*N* = 6). The 19 participants who fulfilled the criteria of either being a woman and/or fulfilled the criteria of displaying autistic traits at T3 and have given their written consent to be contacted again, will therefore be invited. All participants will be contacted by letter, informed about the purpose and content of the study, and asked if they wish to participate in an in-depth interview.

The in-depth individual interviews will be supplemented by two focus group interviews. The participants in this part of the study will be clinicians experienced in assessing and supporting people with autism. The participants will be recruited from local child, adolescent and adult psychiatric out-patient clinics. These focus group interviews offer an additional perspective on the experiences of people with autism, as outlined in objectives 2a and b.

### Data collection

#### Part one: Individual interviews with emergent adults with autism

In-depth semi-structured interviews are described as optimal for collecting data on individuals' personal histories, perspectives, and experiences, particularly when sensitive topics are being explored (59). Building trust, tailoring people's communication needs and preferences is critical to this process (60). Experiences from conducting previous research on interviewing people with autism have informed the basis of the interview guide (42, 61–63). Demographic variables (age, living conditions, level of education, work or social security benefits etc.) were collected at T3, and the interview guide has been designed to update this information. The interview will begin with some concrete questions about age, living conditions and topics of interest to build rapport, followed by open-ended questions in familiar, everyday language. This will ensure both that the participants' common experiences of having autism are captured and provide them with the opportunity to elaborate on topics they find particularly relevant. The interviewer will shift the direction of the issues as new information and insights come to light. The topics in the interview guide, which map on to the objectives described in 1.6, are as follows: (1) Daily occupation and living conditions; (2) Growing up with autism; (3) What is important in life (subjective experience of QoL); and (4) Compensatory strategies. We have included specific probe questions following each main question to allow for elaboration by the participant, where necessary. These probe questions are focused on specific contexts such as school, social relationships, and social situations.

One week before the interview, the main questions from the interview guide will be sent to the participants as an accessible document (including illustrative images, see Supplementary material), allowing them to prepare themselves for the interview. The interview guide will also inform the participants of their rights when participating in research. The interviewer will inform the participants of different ways to communicate the need for a break or the wish to end the interview.

The interviews will be conducted face-to-face by a researcher/clinician (EØ) who is experienced in interviewing people with autism.

#### Part two: Focus group interviews with highly experienced clinicians

We will also conduct two focus group interviews, each with ~4 experienced clinicians, and two researchers as facilitators (EØ and ÅLH). The interviews will start with a presentation of the qualitative findings from the project so far, with a focus on possible contradictions between emergent adults with autisms' descriptions of living with autism, and the clinicians' more general perceptions of what living with autism is like. Subjective experiences of what constitutes QoL for people with autism, how compensatory techniques are used, the ups and downsides of autism, and how autism affects and is affected by their environment, will be discussed. We will explore the perspectives and reflections of the clinicians to understand our participants' lived experiences within a clinical context. The focus groups will offer a dynamic way to capture clinicians' perspectives and, unlike individual interviews, allow for deep discussions between participants and the possibility of identifying points of (dis)agreement.

### Data analysis

All interviews will be audio recorded with the participant's consent, and then transcribed verbatim. All identifiable information will be removed during transcription, leaving the transcribed material anonymized. As noted above, the findings from all (interview and focus group) interviews will be analyzed inductively, using *reflexive* thematic analysis, as this leaves more room for discussing the different perspectives concerning subjectivity and reflexivity from a more social constructivist point of view. At least two researchers (EØ, ÅLH) will conduct the analysis following Braun and Clarke (50) steps, including: (1) familiarization with the data, (2) coding, (3) generating initial themes, (4) developing and reviewing themes, (5) refining, defining and naming themes, and 6) report the findings. NVIVO, a software designed to help organize, analyze, and find insights in interview data, will be used to manage the data. Following the concepts of reflexive thematic analysis described by Braun and Clarke (50, 53), we will code the entire data set. The initial themes will be generated by collecting codes into themes, then developing and reviewing the themes further during the analytic process. As mentioned in section Data analysis, we will analyze the data from a critical realist approach, meaning that we will have to analyze the data in a perspective of trying to identify factors or forces beyond the knowledge conveyed by the participants (51). Also, in part to acknowledge that our own pre-assumptions will influence the way we interpret the data, the researcher conducting the interviews (EØ) will keep a reflexive diary. This will be used to inform discussions during the analysis meetings, and possible elements from the interview context and other perspectives that might influence our interpretations, will also be discussed. The findings will be compared and discussed in analysis meetings with all involved researchers, including representatives from the Norwegian Autism Society. Contradictions and similarities from the individual interviews will be further explored in the expert focus group interviews.

## Community involvement

Representatives from the Norwegian Autism Society have been involved in developing research questions and have given feedback on the interview guide. They advised us to include the use of strategies of everyday routines, and to be aware of motor developmental problems, sensory overload and gender identity issues as possible obstacles in everyday life. One of our co-researchers/authors with autism has also been involved in this qualitative study from the point of grant submission, guiding the research group on the wording and approach to the research questions and interview guide. Representatives of the Autism Society will be present during the analytic process, together with all involved researchers. The Autism Society representatives will be compensated for their contribution.

## Ethics

The study has been approved by the Regional Committee for Medical Research Ethics in South-East Norway (2018/1611/REK Sør-Øst) and the Privacy Ombudsman for research at Innlandet Hospital Trust (IHT) (14173214). The study will be conducted following the Helsinki Declaration of the World Medical Association Assembly. Before each interview, the participant will be informed in detail about the study aims, usage of interview data, participant rights and researcher duties, anonymity, and data storage, before signing the informed consent form specifically related to the study. The interviews will be recorded and encrypted before they are stored on secure servers at IHT. All data analysis will be conducted within the secure environment provided by IHT. This solution follows Norwegian privacy regulations.

The interviews include open questions about the life and functioning of the participants, which means that they might be reminded of difficult and painful memories and experiences. Sensitive topics may be elicited by the conversations within the interviews, although they will not be asked directly about them. Research shows that talking about difficult experiences usually does not contribute to increased psychological distress in participants (64, 65). Nevertheless, to ensure the participants' wellbeing, after each interview, the interviewer will ask if the participant has experienced any distress and will discuss potential concerns directly. If there is a reason for concern, the interviewer will ask for the participant's consent to contact him/her on phone shortly after the interview, for a follow-up talk. If necessary, further arrangements will be made with a clinical psychologist. If any of the participants express general mental health problems throughout the interview, by which they do not receive any support, this will be addressed and the researcher doing the interview will guide the participants to seek support from their general practitioner (GP) or other local mental health services.

## Dissemination

Findings will be presented at national and international conferences and published in open access peer-reviewed autism journals. We will further distribute our reflections upon the findings to different clinical institutions where knowledge about autism is conveyed. Distribution will also be done through other channels, such as the media, social media, patient interest groups and organizations. Scientific lectures will be held at different teaching institutions and treatment clinics. To reach the autistic community, the findings will be shared with the Autism Society at national and local meetings, in their membership magazine and on their social media channel.

## Ethics statement

The studies involving human participants were reviewed and approved by the Regional Committee for Medical Research Ethics in South-East Norway (2018/1611/REK Sør-Øst) and the Privacy Ombudsman for research at Innlandet Hospital Trust (IHT) (14173214). The patients/participants provided their written informed consent to participate in this study.

## Author contributions

EØ wrote the manuscript, based on the project plan written by all the other authors (ÅH, SO, EP, MØ, ES, and PA). All authors revised, commented on, and accepted the final manuscript.

## Funding

This study is supported by grants from Innlandet Hospital Trust, project Numbers 150663, 150670, and 150648.

## Conflict of interest

The authors declare that the research was conducted in the absence of any commercial or financial relationships that could be construed as a potential conflict of interest.

## Publisher's note

All claims expressed in this article are solely those of the authors and do not necessarily represent those of their affiliated organizations, or those of the publisher, the editors and the reviewers. Any product that may be evaluated in this article, or claim that may be made by its manufacturer, is not guaranteed or endorsed by the publisher.
