# Supplementary material for: Exploring life with autism: Quality of Life, daily functioning and compensatory strategies from childhood to emerging adulthood: A qualitative study protocol
Source: Front Psychiatry. 2022 Nov 25;13:1058601. doi: 10.3389/fpsyt.2022.1058601 (PMC9732257; doi:10.3389/fpsyt.2022.1058601)

Questions about you

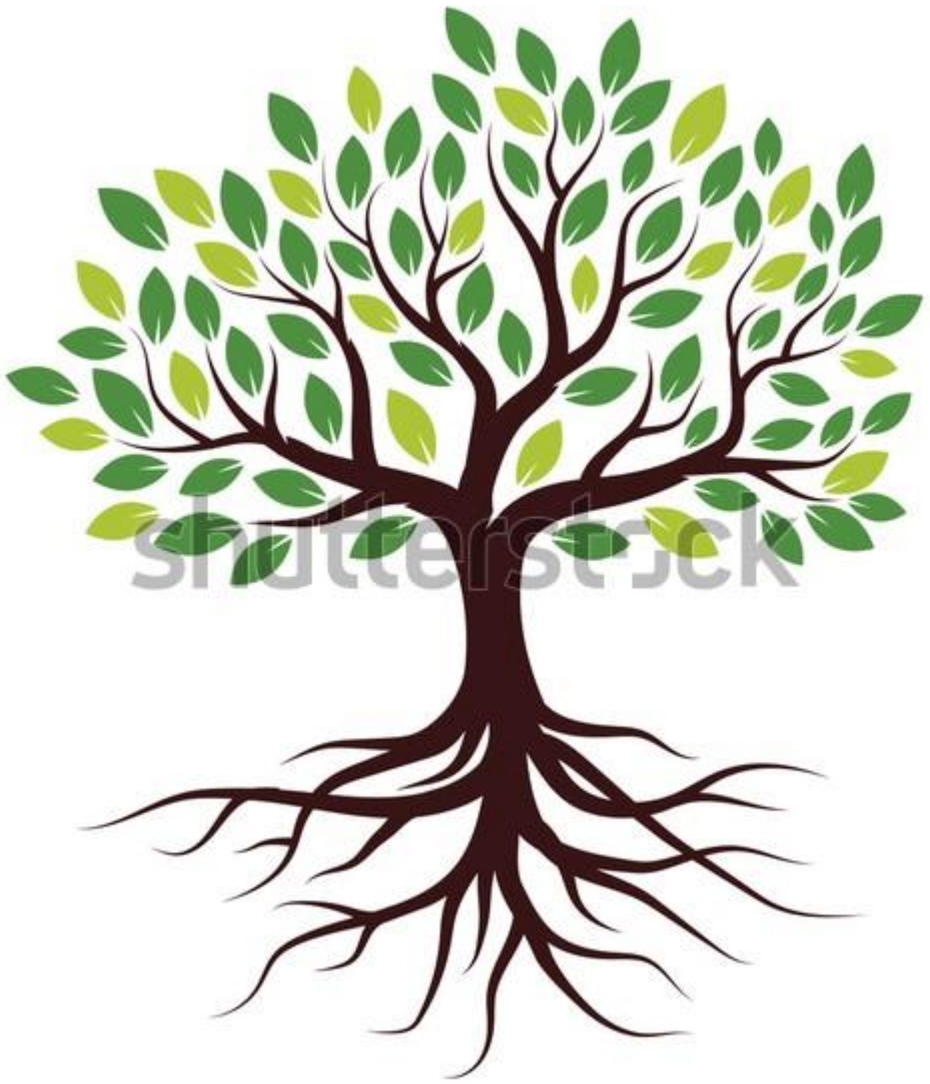

1. Tell me a little bit about yourself

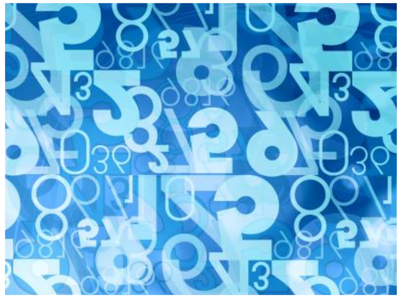

How old are you?

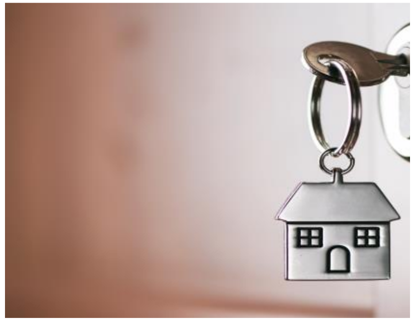

Who do you live with?

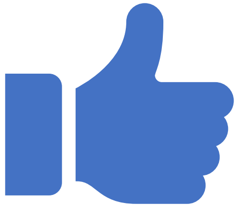

What is your favourite thing to do?

2. What has it been like to grow up with autism/Asperger syndrome?

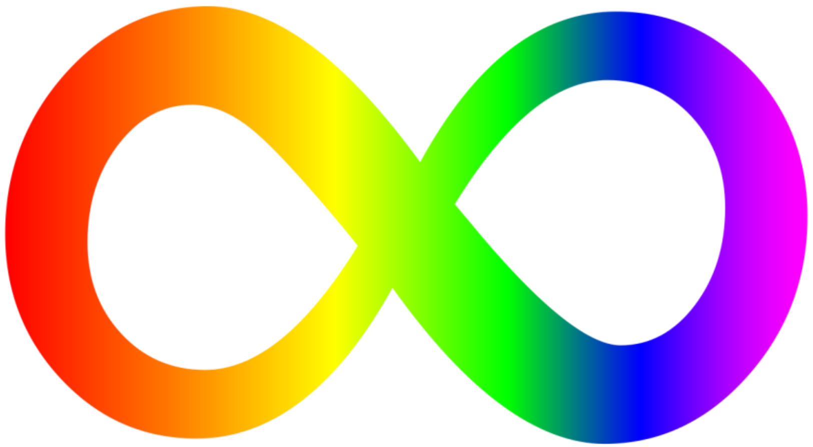

3. What is important to you, in order to have a good life?

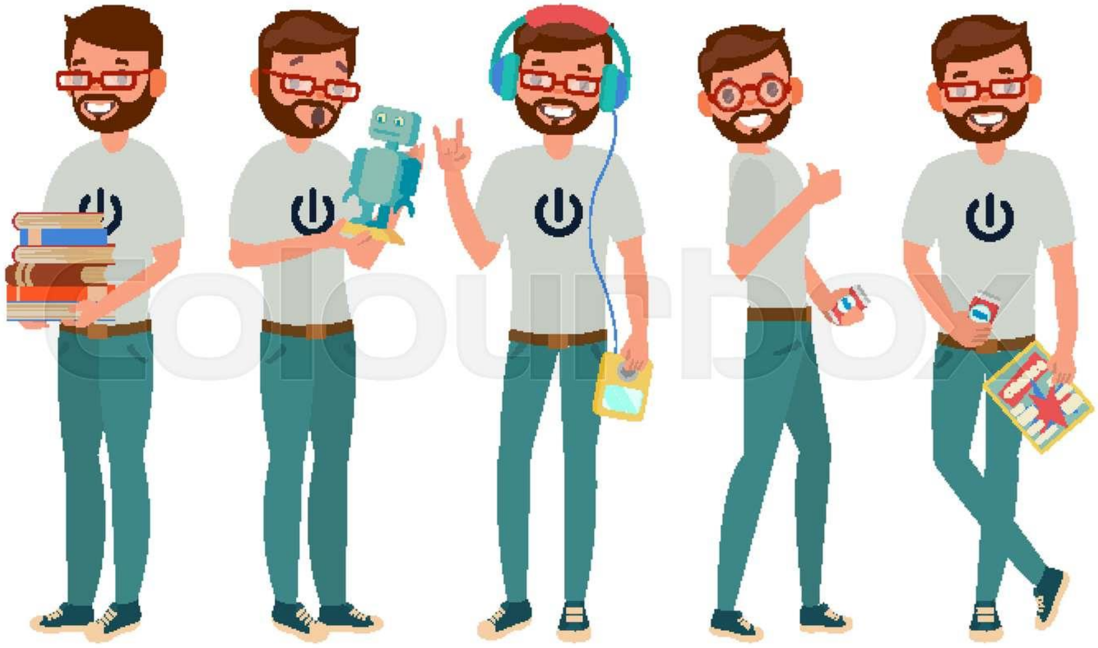

4. Do you sometimes behave in certain ways to be as similar to others as possible?

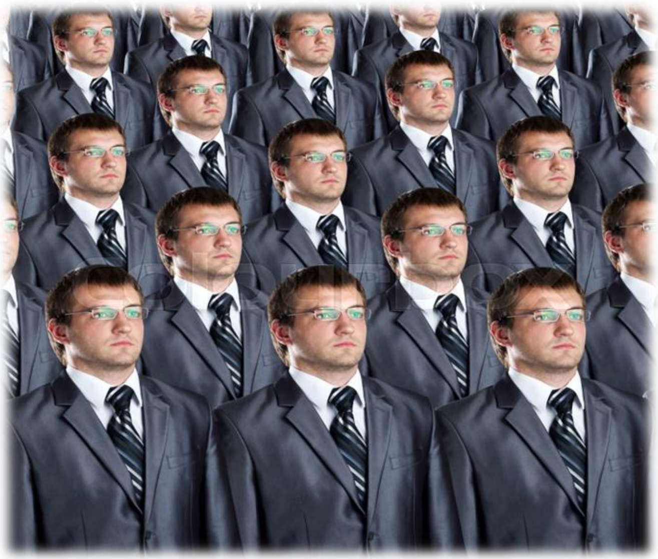

5. Thinking about the next five years, how do you picture your future when it comes to:

- friends
- studies
- work
- love life
- establishing your own familiy

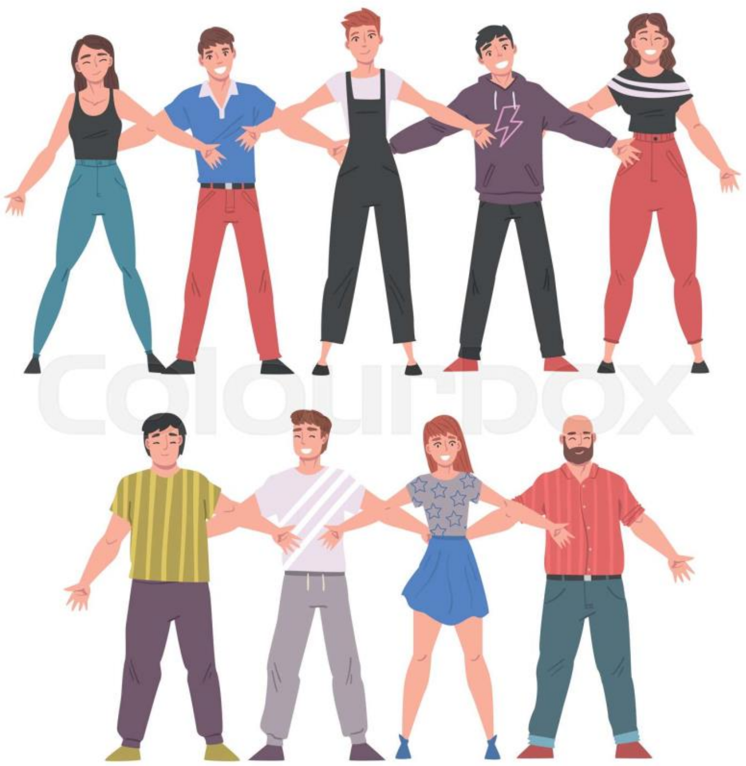

6. What are you grateful for in life?

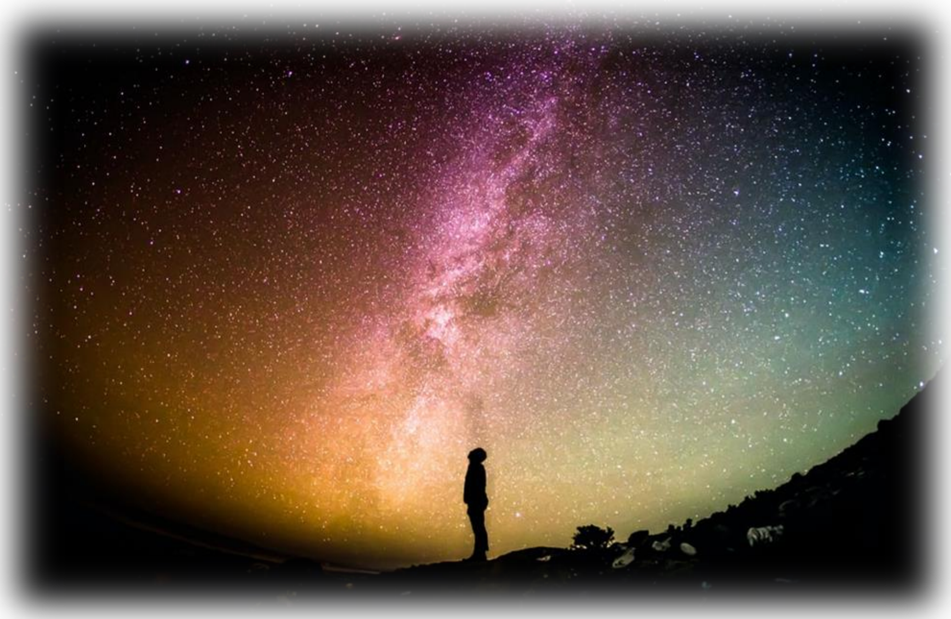

Supplement: Supplementary file 1 [file Data_Sheet_1.PDF]
