# Supplementary material for: Exploring life with autism: Quality of Life, daily functioning and compensatory strategies from childhood to emerging adulthood: A qualitative study protocol
Source: Front Psychiatry. 2022 Nov 25;13:1058601. doi: 10.3389/fpsyt.2022.1058601 (PMC9732257; doi:10.3389/fpsyt.2022.1058601)
Supplement: Supplementary file 2 [file Data_Sheet_2.PDF]

Interview guide "Exploring life with Autism"

Thank you for participating in this project. I am interested in getting to know how it has been for you to grow up with autism/Asperger syndrome, about your life in the previous years, concerning family, friends, leisure time and school or work. You will get the opportunity to talk about issues that are important to you, too.

There are no right and wrong answers. I am just very interested in how it is to live your life. I hope this will be a good experience for you. If something gets difficult for you during the interview, it is important that you tell me, so that we can take a break or stop the interview.

I will record this interview. This is to help me to register everything you say. I will listen to the tape afterwards and write down exactly what you have said.

I know that some people with autism/Asperger can feel uncomfortable about eye-contact. Where do you want me to look when we talk?

Do you have any questions before we start?

1. **Tell me a little bit about yourself; How old are you? Who do you live with? And what's your favorite thing to do?**

Probe questions:

- *What are you good at?*
- *Do you spend a lot of time with your family? What do you like to do with them?*
- *Do you study or work, and what do you think of that?*
- *Do you spend time with others through studies/work, leisure activities, online games etc? Tell me about that.*

2. **What has it been like to grow up with autism/Asperger syndrome?**

Probe questions:

- *Tell me about how it was like to be diagnosed.*
- *Do you talk about autism in your family? How does your family tend to talk about it?*
- *How important is being autistic (having autism/Asperger syndrome?) to you?*
- *Do you tend to tell people that you're autistic/on the spectrum? Friends, or other people you interact with? Do they understand what that means? How do they react to it?*
- *Have you experienced that others treat you different because of your autism?*
- *Can you think of any specific advantages with having autism/Asperger syndrome?*

- *How was it like for you to go to school? What did you enjoy at school? What did you find challenging at school?*
- *What kind of support have you received over the years? From therapists, teachers, other people at school or at home? Has that support been helpful?*
- *Have you developed any of your own strategies to deal with issues that were previously challenging for you?*

3. What is important to you, in order to have a good life?

Probe questions:

- *What makes you happy? e.g., friends, family, interests, passions, talents.*

- *What things do you find challenging? This could be connected to studies/work, making decisions, getting out and about, economy, difficult feelings, sensory issues, focussing long enough, initiate tasks, find your own identity.*

- *Are there things you could do to make you happier/your life easier? What are they? What would help you right now to achieve these things?*

- *Can you think of anything that others could do to help you get a better life?*

- How do you feel now, compared to a couple of years ago?

- To make decisions in your life, what does that mean to you? What do you decide for yourself, and what do others decide?

4. Do you sometimes behave in certain ways to be as similar to others as possible? Tell me about those times. How do you feel about that?

Probe questions:

- *Are there times where you think that behaving in certain ways can be helpful?*
- *When is it not-so-helpful?*
- *Do you sometimes expect others to behave in certain ways to adapt to your needs? Can you give me some examples? How do you feel about that?*
- *Are you ever in the company of people where you don't have to think about what you say and do?*
- *Some people worry a lot about whether they have done things the right way or not. Is this something you worry about?*
- *Are there things the people you work with/go to school with could change to make your life easier/better? What kinds of things?*

5. ***Thinking about the next five years, how do you picture your future when it comes to***

- *friends*
- *school*
- *work*
- *love life/ establishing your own family*

Probe questions:

- *Do you wish for a boyfriend/girlfriend?*
- *…. Or would you like to live different than you do now, or how others live? How?*

6. ***What are you grateful for in life?***

That's it! That's all my questions. Thanks so much for speaking with me and for sharing your experiences – it was wonderful to be able to hear it. I hope that you are feeling ok about it too.

Regarding next steps, once we have finished speaking to everyone, we will put everything together and write a summary that tells you what we did, what we found and what it all means.

Finally, I hope that you enjoyed our conversation. But if you feel worried or upset as a result of what we've spoken about today, I can give you the details of some places to contact if you need support.
